# Gynæcology in Japan

**Published:** 1877-05

**Authors:** A. Wernich

**Affiliations:** Jeddo


### Translations.

### GYNÆCOLOGY IN JAPAN.

By A. WERNICH, of Jeddo.

[Translated by Dr. James I. Tucker, Chicago.]

It seems to me that in view of the prejudices and the false impressions which have been conveyed by those writers who claim to portray the peculiarities of the Japanese people, especially, the women of Japan, I am justified in communicating my own impressions to the circle of cultured readers who are especially interested in gynæcology.

Since my arrival, in Nov. 1874, a little gynæcological department has been fitted up here, consisting of ten or twelve beds. This is the first thing of the kind in Japan, and it is my great ambition to keep it constantly filled. But a small number of the patients who come to the Dispensary (*Poliklinik*) are able to conquer their aversion to submitting themselves to an examination by many different persons for the purpose of instruction. Consequently, the material, even including the cases derived from private practice, is at best nothing to boast of. All information must be obtained by means of an interpreter, and by this round-about method, not only much time is lost, but it is rendered very difficult to get at the complicated physical and mental conditions of the patient.

I send you a brief description of the physique, habits and constitution of the women, as an introduction to a communication concerning menstruation and female diseases.

The Japanese women are not beautiful, though much may be said to their praise.

No one would be willing, however, to say seriously that he had ever seen in the whole country a really perfect, symmetrically built, nobly-formed woman. I abstain here from a description of all mere outside appearances, such as the thick hair, resembling a horse's mane, whose braids (not curls) are held in place by the free use of fats, generally oil somewhat rancid; the impure complection, which they conceal by means of powder applied to the neck, chin and cheeks, but which, nevertheless, presents a dirty, grayish-yellow appearance upon the forehead and at the borders of the hair; the scar of moxæ also, and other popular remedies, on the back and breast. I refer especially to the structure of their bodies. If your ideal of female beauty be Juno, Venus or Hebe—it is wholly unknown in Japan. One is bony, and has a neck like an ox; another has hanging breasts and frightfully broad hips; a third is lean and lank, with withered arms, sunken abdomen, and prominent pelvic bones; but all three would banish all delusion by the characteristic Japanese crooked legs. The admirers of the women of this country praise the round, graceful form of the neck and shoulders; and it is not to be denied that the not ungraceful lines of these parts, which proceed from the moderate deposit of adipose tissue, together with the velvet-like softness of the skin, and somewhat pleasing color make a happy combination. With the neck and arms, the *danseuse* makes her graceful movements, while her legs under the long garments, are seldom visible.

The gait is as ungraceful as possible. They shove one foot along after the other, in their stilt-like, horribly slippery and noisy wooden shoes, which the woman of the better class wears on the street, as well as the peasant girls, with crooked knees and prominent belly, the rest of the body being bent backwards; only the aspect of the upper part of the body sometimes carried with graceful ease, gives some relief to the offended eye and ear. This is equally true of the unmarried and married women of fashion. I need not mention that those women who adhere to the custom of shaving the eyebrows and blackening the teeth do not present a particularly charming appearance.

The pelvis is broad and very capacious. A pelvic deformity, in which the pelvis is generally narrowed, I have seen but once, as a congenital defect. The symphysis forms a very large obtuse angle. I do not recollect among my cases to have seen a narrowing of the introitus vaginæ. The growth of hair upon the mons veneris is in direct contrast to that upon the head, and the thickness of the individual hairs is difficient; remarkably seldom it forms a triangle, having an oval contour corresponding to the vulva. The labia are lean, and even in young persons, very flabby.

The vagina is short; I never found one over 7 cm. long. I have never seen a hymen. As a general thing the perinæum seemed to be not particularly broad—congestion and tumefaction of the portio vaginalis was a matter of as frequent remark in my examinations, as in European women; it did not escape even the observation of Japanese physicians and interpreters.

According to unscrupulous travelers who have written about the habits and customs of the people, and who have derived their information from brothel experience at Nagasaki or in hospitable taverns of Kanagawa, the Japanese women are regardless of propriety, and destitute of shame. I am glad to say that in this respect I share the opinion of so distinguished a writer as Mitford, who has resided here many years; the author of "Tales of Old Japan" has done full and deserved justice to the Japanese ladies of patrician and plebian ranks, who are not prostitutes. Even the waiting-girls of respectable tea-houses are unapproachable to strangers, and old experienced suitors do not forget their unsuccessful efforts to seduce this or that tantalizing dancing-girl. The stories about guardians and relatives abandoning women to indiscriminate prostitution rest upon misunderstanding or brag. The numerous contracts made between Japanese maidens and their European neighbors are the best evidences of it. It is the legalization of a custom, which, according to our ideas, involves an immoral relation, that has given to these women the unenviable reputation of being frivolous and loose. Every marriage may be dissolved; it is a contract holding in force a few years only. He who has kept his wife for this length of time, or longer, seldom abandons her. If she is rendered useless by sickness or debility, so as to be unfit for sexual intercourse, she provides for her husband one or more concubines, and it is understood as a matter of course that the man shall possess every means necessary to satisfy his animal passions. All these women assist one another in the duties of the household—an admirable provision in view of the natural indolence of the Japanese women. The first always officiates as house-keeper, and can then expect only acquiesence on the part of another, who has borne a son, because she herself has not been able likewise to provide for the family. The unmarried, but not abandoned Japanese woman of ordinary standing, always demands from the foreigner who troubles himself about her, a sort of marriage contract, for a month. However different this month-marriage may seem from a real marriage, we should not underestimate its significance. If any one is inclined to take a different view of the matter, he should bear in mind that this marriage relation is legitimate, and that the woman is, for the time being, really the wife of the stranger. To the fullest extent she takes possession of his house, bag and baggage, arranges her allotted apartments in all their details, and does not fail with the universal skillfulness of woman, to adapt herself to her new circumstances, and to make her function very important. She stands in an arbitrary relation to her domestics; she is obliged to pay a small tribute (generally 15 Rio=60 German marks) from her income to every one in the house, and therefore each one likes her and renders her assistance in all things. Then these persons endeavor to make themselves indispensable; they attend to every little affair, they satisfy every whim of the lord of the household, they provide for his comfort; in short, they are loyal in the highest degree in order that their term of service may extend beyond the limits of the appointed month.

In this way, attachments are formed, which, springing from a change of views, and cooling down of passion, become at last enduring. I am acquainted with many Europeans who have lived with their Japanese companions six and eight years, and who have given up all idea of dissolving the marriage relation and of returning home. And yet I have been speaking thus far only of maidens of low degree. A family which prides itself upon its respectability, sometimes, it is true, will give its daughters to a foreigner, but only under the express condition that she is given him for a definite period of time, while he is in the country, and that she is not to be taken out of the country.

In one thing observers agree, viz., that these so-called "fast" women, in most cases, are thoroughly constant. In point of attachment experiences differ. This much is, however, certain, that in respect to food and clothing they come far short of the requirements of the European. They enjoy only Japanese food, which they prepare for themselves; even in respect to drinks, cake, dainties and the like, there is no commonality of taste; neither are they capable of being educated to our mode of living. Watches and rings are the only trinkets which they possess and highly prize. From all other foreign notions they stubbornly abstain. Yet after years of living together, the Japanese women do learn from the European to make use of a pillow, made of the makura. These are made so the back of the head hangs free, in order that the hair may not become disheveled during sleep. But in clothing they will brook no change. The cloth bound about the hips in the form of a little under-garment, three or four folds over one another, like a German dressing-gown, which is fastened by as many smaller girdles, and even the foot wide upper garment, (obi) with which we are already familiar, through illustration, are clung to with pertinacity, in spite of the greatest love for the foreigner. In customs the women are more conservative than the men.

That, which, despite all this incongruity, gives them the power, in many cases, to fascinate the men, has doubtless given them in part their bad reputation. As many observers assure us, they will not submit themselves to the man whom they have once loved and left. How much this is due to natural instinct, or to a feeling of shame, I am not prepared to decide. At all events, I hold to the opinion, after many opportunities to observe, that the last reason is not the predominating one.

And now I come to my own experience which I have gathered from a large number of gynæcological examinations. The Japanese certainly do not regard the naked body as something unclean, and no one will now anxiously veil his legs and his breast. The greatest care however is taken to prevent the exposure of the genitals, which are guarded with the fullest consciousness of shame. Every male patient, if he has wholly disrobed himself for the purpose of examination, still holds the little T-bandage-like cloths (Fundusi) about his hips; every woman hesitates in the most natural way to lift the little wrapper or girdle, and yields only when she is shown the medical necessity. I have seen women weep bitterly for shame when treated by the assistant physicians with undue regard to their feelings, and frequently I have been requested not to push my examinations of a woman in confinement, or with an abdominal tumor.

With reluctance the mother of a half-clad child uncovered it for the purpose of examining its chest for pulmonary disease, and I could not get one-fifth of the female patients who were to be placed upon the table in the gynæcological clinic to come into the hall where the students were present, without being first wholly concealed.

They have apparently great confidence in the physician; delicacy and prudence, particularly in hysterical persons, develop the same foolishness and deception that we observe in half-educated European women under the same circumstances. The women exhibit a degree of fortitude under the discomfiture and pain produced by minor operations and frequent examinations, and know very well how to restrain themselves. As a rule it is difficult for them to master a certain degree of sexual erethism, which happens to them more frequently than to our women undergoing like examinations; and on account of the erection of the clitoris, of the portio vaginalis, they readily secrete the glairy cervical mucus, and give expression to the well-known signs of sexual excitement, hurried respiration and disturbed utterance.

Yet this excitement, with most of them, became less and less upon repeated examination. The number who attend our gynæcological clinic and the dispensary has steadily increased during the course of the last winter, so that the department will be enlarged in the new hospital building, which will soon be ready.

With characteristic Japanese politeness, the patients always accepted willingly the chosen local appliances for treatment, (injections, tampons, suppositories, pessaries, hip baths, etc.,) and said that they had followed the directions with great precision. I am obliged to doubt this exactness in many cases, in accordance with those who have had much experience with the Japanese character, yet I believe I should restrict my doubts to the minority.

The weak and tardy reaction which forms the dominant feature in the physical life of the Japanese, and impresses upon almost all their sicknesses a relapsing and somewhat protracted course, has often taxed our patience in gynæcological cases. Yet we cannot affirm that the women, on account of their particular anomalies of constitution in this respect, are worse than the men.

Confined to the narrow limits of the house, and the family knowing nothing of providing food, scarcely thinking of their own advancement and an independent mode of life, not yet susceptible to the allurements of passion, the Japanese maiden awaits with the utmost resignation the period of the first menstruation, and lives in the manner several years more, in order, as soon as an opportunity offers, to unite her destinies with a suitable man; it may be for a limited time, it may be for life. Hinderances to marriage in the modern romantic sense, do not exist; but as long as she still lives in the house of the man who feeds and clothes her, she must be true to him. For several years the husband, who may discover inconstancy in his wife, has had the right to put her to death without ceremony. Among the eight reasons on account of which a marriage may be laid aside, besides the expiration of the term, and after years of living together, figure especially physical defect, (for instance, lepra, stinking breath,) loathsome skin-disease (herpes and the like).

As mothers, the Japanese are unweariedly devoted to their children, from an instinctive affection. As an instance in common domestic life, may be mentioned the continual carrying about of the child upon the back in the dress of the mother, so that among twenty who are attending to the duties of the house, certainly we see fifteen in their common gown, skipping about with their child, for all the world as if a parasite were upon the mother. If there are older children, one carries the other upon the back (Umbo), so that considering the youth of the bearer, we are often in doubt which head belongs to it and which to the little brother or sister. Hysteria exists even in an aggravated degree, but we are led to believe no less frequently in the feminine than in masculine sex. Spasms are rare as well as contractions and convulsions of single muscles and limbs, also the laughing and weeping paroxysms which in my experience have seldom occurred. This hysteria is quite exceptionably referable to real trouble in the sexual organs. The great majority of hysterical Japanese are among the married women, who have passed through one or more confinements. Older persons, too, at the cessation of menstruation, become hysterical.

The Japanese women are in general very fruitful. The houses would probably be more noisy with children, were it not for long nursing and abortion. The foreigner, who makes a Japanese woman his concubine, declares in very many cases that he does not wish to have children. It is left with her, however, whether she will fulfill his wishes or not. The mixed breed, which came under my observation, made a very pleasing impression. I remember particularly several little Franco-Japanese that looked like pretty little French children. These children for the most part remain in the country, and alas! become accustomed to European clothing and education.

They are treated with indulgence and respect, though they are scrutinized by every genuine native of Japan. And certainly they find their way back with difficulty to re-adoption of the uncommonly unassuming manners of the natives. After the departure of the father, the mother returns into the circle of her family, but after that she is seldom taken to wife by a Japanese.

After these general observations, I come to speak next of

MENSTRUATION.

The menses begin in the 14th or 15th year and do not cease until about the end of the 40th year. In reference to this fact, there is a play upon numbers of Chinese origin, as follows: In woman the power of reproduction begins at 7+7 and ends at 7x7; in man it begins at 8+8 and ceases at 8x8. Fewer persons menstruate very early than very late, and the beginning of the period before the 12th year of age, belongs to the remarkable occurrences. Girls, whose menstruation is tardy, (occurring in the 18th year), are not generally sick—still more rarely anæmic in the sense in which we employ the word—but they are simply backward in evolution, remain artless like children. "They do not trouble themselves about hairpins and the artistic toupee," said my interpreter. They do not powder their necks nor put on the girdle of the young woman, but dress and expose themselves like children, play with the boys upon the street, etc. Their physical and mental development is somewhat anomalous. They remain angular, while on the other hand the young Japanese woman who has undergone the menstrual evolution, generally becomes athletic, her breasts and hips become extraordinarily broad. Regarding the amount of menstrual flow, it would, perhaps, be impossible to arrive at a correct estimate, as we do at home, after observation extending over a number of years. There is a very peculiar arrangement, which the woman makes use of during menstruation, and which controls to some extent the amount of the flow. She puts on the usual well-constructed T-bandage, which is called *kama*. But this is not sufficient to absorb the fluid. Something more is necessary. The people of the Orient are so rigidly observant of the rules of cleanliness that they never use a second time the clothing and pieces of cloth which have once been soiled with the fluids of the body—blood, pus, and discharges from the nasal and bronchial mucous membrane. This fact is well-known from the description which writers give of the Japanese and Chinese handkerchiefs made of paper. In the higher ranks of society, the menstrual excretion is considered unclean, and consequently paper is used for its absorption and removal. From the abundant store of paper-leaves which are used for various purposes, the women squeeze pledgets into the shape of balls of the size of almonds or walnuts, and stuff these into the vagina according to its necessities. A woman, who, during this period, visits for example the theatre, resorts to this procedure several times in the privy. She knows quite exactly when these tampons which she has introduced have soaked up the blood and then squeezes up a new one. In severe cases of *fluor albus*, I have sometimes found these paper-balls in the vagina. The number of these tampons which is introduced corresponds to the duration and abundance of the product of menstruation. It varies from six to twelve.* A short menstruation is regarded as a sign of good health; much less stress is laid upon consistence, color and foreign admixture.

In healthy women menstruation lasts three to four days; in hospitals, in different pathological conditions, naturally longer. A popular song, not altogether decent, in which the maiden consoles her lover, to the effect that he must content himself during this period without normal sexual enjoyment, fixes the time at seven days. This reckoning has been carefully observed, as it is valuable in diagnosis to notice any shortening of the stage of menstruation, as well as the length of the interval. Physiological menstruation is attended by light, labor-like pains in the abdomen, and some sense of pressure in the temples. Pain and sensation of cold in the loins, drawing-sensations in the thighs, pains in the back of the head, and in the forehead, are well-known pathological signs.

As an emmenagogue (not purgative), an infusion of the root of *rubia cordiflora*, is used, which the women call *shenkong akane*. Yet of late years preparations of iron and cinchona, pediluvia and sinapisms, have gained popular favor. Sometimes mustard and capsicum are used internally. Cessation of the menses is considered by the Japanese women the most reliable point from which to reckon pregnancy. When the year was officially divided, as formerly, into lunar months, it was still easier, as all she had to do was to reckon ten such periods of time before confinement. Singular enough she is perplexed if the last menstruation continues from the last day of a (calendar) month to the first of the next (*zéki matangi*), as the technical expression is. The reckoning is in-

* In some of the provinces paper wadding is used.

exact, for she counts in the month that has begun as a whole month. She makes a miscount in this manner.

The women and the physicians are tolerably well acquainted with other things which may cause a temporary amenorrhœa. That acute diseases, attended by fever as well as radical abortions, may be followed by this as a consequence, is universally accepted. The relation of menstruation to lactation has been the subject of careful medical observation. I was told that "if a woman has never been pregnant before, lactation may last five years; it extends into the fourth year as a regular thing, even when the child does not depend exclusively upon the mother's breast for nourishment. Yet the milk is not abundant longer than three years. Menstruation regularly reappears during this long period of lactation; yet it is regarded as unusual for it to reappear prior to three months after confinement. Return of the menses is not thought to exert an influence upon the quantity and quality of the milk. If menstruation occurs but once, and does not re-occur, and if lactation ceases gradually 2 to 3 months later, we can decide without the possibility of being deceived, that there is a new conception. After this time (2 to 3 months) the milk invariably dries up."

Alterations which are produced by the menopause, are as follows: improvement of the health in weakly women; development of catarrh of the stomach and bowels; obesity.

During her "period," a woman is always forbidden to bathe. Coitus and hard work are also forbidden. She fears taking cold very much, and calls it characteristically *shimokase* (wind from below.) In some provinces of the interior, especially in Hida, the women are very earnestly advised, during this time, to relinquish visiting the temple, and prayer to the gods, and to good spirits; in other provinces they are obliged to spend the time in their apartment, and not allowed to eat with their families. As soon as a woman finds that the flow has ceased, she takes a bath, puts on other clothing, and lays aside the T-bandage. The maiden early becomes acquainted with these rules by listening to the conversation of other women, young and old. Formerly the pernicious custom prevailed among old female servants and aunts, of giving secretly to the daughters of distinguished families, in whose presence nothing had ever been said about *hatzubana* (first blood), books which contained an accurate description of menstruation, and at the same time obscene allusions. At the present time traffic in this kind of literary product is forbidden.

It was very interesting to me to find, upon translating many of the expressions for "menstruation," none which conveyed the idea that menstruation is an act of purification. The Japanese consequently regard this blood as in the highest degree unclean—perhaps the most unclean of the excretions—but not, that they are thereby cleansed. The commonest term is *gek-ke*, which signifies simply the monthly rule. *Mengori* or *megori*, the next most in use, and also a somewhat finer term, is, literally translated, a thing which moves in a circle, or one which regularly returns. *Akane son-ke*—a somewhat ordinary expression used in popular songs, and by wits, means "red-color; "geschim" means monthly messenger, or announcement, and "jakh" means simply, duty. The two last have fallen already somewhat in disuse.

[TO BE CONTINNED.]

